# Therapeutic effects and mechanisms of Fufang Longdan mixture on metabolic syndrome with psoriasis via miR-29a-5p/IGF-1R axis

**DOI:** 10.3389/fphar.2025.1585369

**Published:** 2025-05-09

**Authors:** Guangyun Luo, Xiangyi Kong, Fang Wang, Zhiming Wang, Zhuo Zhang, Huan Cui, Yiwen Zhang, Wen Huang, Xuesong Yang, Jianzhou Ye

**Affiliations:** ^1^ The First Clinical College of Nanjing University of Chinese Medicine, Nanjing, Jiangsu, China; ^2^ College of Basic Medicine, Yunnan University of Chinese Medicine, Kunming, Yunnan, China; ^3^ Department of Dermatology, First Affiliated Hospital of Yunnan University of Chinese Medicine, Kunming, Yunnan, China

**Keywords:** metabolic syndrome-psoriasis comorbidity, Fufang Longdan mixture, MiR-29a-5p, IGF-1R, traditional Chinese medicine

## Abstract

**Background:**

The occurrence of comorbid metabolic syndrome and psoriasis (MS-P) is owing to the complex interplay between metabolic dysregulation and inflammatory responses. However, current treatments have shown limited efficacy in improving the symptoms of both conditions simultaneously.

**Objective:**

This study aimed to investigate the therapeutic efficacy of Fufang Longdan Mixture (FLM) in treating MS-P comorbidity, elucidate its mechanism through the miR-29a-5p/IGF-1R axis and evaluate treatment responses between APOE^−/−^ and C57BL/6 mice.

**Methods:**

UPLC-Q-exactive-MS/MS analysis was used to characterise FLM’s chemical composition. Metabolic syndrome was induced in APOE^−/−^ and C57BL/6 mice using a high-fat, high-sugar diet, while psoriasis-like lesions were induced in the mice via the administration of imiquimod. The mice were randomised into control, model, Yinxieling (8 g/kg/d) and FLM (0.5 mL/d) groups. We assessed the treatment efficacy through metabolic parameters, hematoxylin and eosin (H&E) staining and inflammatory cytokine profiling. The direct targeting of IGF-1R by miR-29a-5p was verified via dual-luciferase reporter assays. We analysed the expression patterns and interactions of miR-29a-5p/IGF-1R using RT-qPCR, Western blotting and fluorescence *in situ* hybridisation.

**Results:**

Chemical analysis identified 2,665 compounds in FLM, which were predominantly shikimates and phenylpropanoids (32%), alkaloids (20%) and terpenoids (13%). FLM significantly improved metabolic parameters in MS-P mice, including fasting glucose levels, insulin resistance indices and lipid profiles (p < 0.05), with more pronounced effects observed in the C57BL/6 mice (p < 0.05). FLM demonstrated superior metabolic regulatory effects compared with Yinxieling (p < 0.05). The treatment significantly reduced Psoriasis Area and Severity Index (PASI) scores and inhibited epidermal hyperplasia (p < 0.05). Furthermore, FLM suppressed the pro-inflammatory cytokines, such as GM-CSF, IFN-γ, IL-9 and IL-17, while elevating the anti-inflammatory IL-10 levels (p < 0.05). Dual-luciferase assays confirmed that IGF-1R is a direct target of miR-29a-5p. Mechanistic studies revealed that FLM upregulated miR-29a-5p expression while downregulating IGF-1R (p < 0.05), with evident co-localisation in lesional tissues.

**Conclusion:**

Our findings demonstrate that FLM effectively ameliorates MS-P comorbidity through modulation of the miR-29a-5p/IGF-1R axis, showing significant therapeutic efficacy across different genetic backgrounds.

## 1 Introduction

Metabolic syndrome (MS) comprises a cluster of metabolic disorders characterised by insulin resistance, hypertension, dyslipidemia and abdominal obesity (Neeland et al., 2024). Recent studies have revealed strong associations between MS and various chronic inflammatory diseases, with a notably increasing comorbidity rate with psoriasis (Colca and Scherer, 2022). Psoriasis, an immune-mediated chronic inflammatory skin disease, affects over 60 million adults and children globally (Bu et al., 2022; Griffiths et al., 2021). In addition to abnormal keratinocyte proliferation, this condition is closely linked to systemic metabolic dysfunction (Gisondi et al., 2018). Meta-analyses have shown that patients with psoriasis exhibit a 2.02-fold higher risk of developing MS compared with the general population, suggesting shared pathological mechanisms between these conditions (Qiao et al., 2021).

Molecular pathological studies indicate that the mechanism of metabolic syndrome-psoriasis (MS-P) comorbidity involves complex metabolic-inflammatory network dysregulation (Metin et al., 2023). This disorder manifests through interactions between metabolic abnormalities and immune imbalance, coupled with epigenetic regulatory changes (Ikeda et al., 2022; Niedźwiedź et al., 2024). Recent research has highlighted the crucial role of microRNA-mediated epigenetic regulation in both metabolic and inflammatory diseases. The miR-29 family, a highly conserved group of microRNAs, shows significant expression changes across various pathological processes (Dalgaard et al., 2022; Kansal et al., 2022). Studies have demonstrated that miR-29a levels in the peripheral blood of patients with type 2 diabetes correlate negatively with insulin resistance indices (Massart et al., 2017). Under hyperglycemic conditions, miR-29a participates in metabolic stress responses by regulating the IGF-1 signalling pathway (Dini et al., 2021). In skin lesions, miR-29a also demonstrates significant regulatory functions (Ge et al., 2019; Kumar et al., 2024). While miR-29a has been separately investigated in both metabolic dysregulation and skin pathologies, its specific role as an integrative molecular mediator in the MS-P comorbidity context remains unexplored. The concurrent dysregulation patterns observed in these separate conditions suggest that miR-29a may function at the intersection of metabolic-inflammatory networks. Its documented capacity to regulate both insulin sensitivity and inflammatory processes positions miR-29a as a potential mechanistic link between systemic metabolic disturbances and localized cutaneous inflammation. This dual regulatory capability makes miR-29a a compelling candidate for further investigation in the context of MS-P comorbidity.

IGF-1R, a key receptor in the insulin signalling pathway, plays a central role in metabolic homeostasis and cell proliferation regulation (Vogt and Hobert, 2023). Previous research has shown that abnormal IGF-1R activation not only contributes to insulin resistance but also promotes cell proliferation via the PI3K/AKT pathway (Guo et al., 2021). While TargetScan database analysis predicts IGF-1R as a potential miR-29a target, experimental validation of their direct regulatory relationship is necessary. Based on the previous findings, we hypothesise that in MS-P pathology, miR-29a may participate in the interaction network between metabolic disorders and epidermal hyperproliferation by regulating multiple target genes, including IGF-1R.

Current clinical treatment strategies for MS-P comorbidity primarily focus on controlling the symptoms of both conditions separately. For MS, treatment typically involves glucose-lowering and lipid-regulating therapies (Persson and Bondke Persson, 2018). In mild cases of psoriasis, patients usually receive topical glucocorticoids or vitamin D3 analogues, whereas moderate to severe cases require biological agents such as IL-17/IL-23 inhibitors (Thomas et al., 2024; Wang et al., 2023). However, these treatment approaches have significant limitations. Research data indicate that anti-IL-17 therapy may abnormally elevate fasting glucose levels in patients with MS, whereas traditional immunosuppressants such as methotrexate may exacerbate hepatic steatosis (Bauer et al., 2017; Lin L. et al., 2022). Therefore, the development of therapeutic strategies that simultaneously address both metabolic regulation and anti-inflammatory effects holds significant clinical value.

Fufang Longdan Mixture (FLM), developed by the Yunnan Provincial Hospital of Traditional Chinese Medicine, showed significant efficacy in previous clinical observational studies for dermatitis and eczema (Huang et al., 2010; Luo et al., 2019). In the study by Huang et al., 68 patients with various types of dermatitis and eczema were treated with FLM, resulting in an overall effective rate of 73.53%. After 2 weeks of treatment, patients demonstrated reduced pruritus, improved erythema, papules and scales, and decreased lesional areas. Mechanistic studies revealed that its active components exert therapeutic effects through multiple pathways. Gentiopicroside ameliorates psoriasis-like lesions by inhibiting keratinocyte activation through the Keap1-Nrf2 pathway and addresses metabolic abnormalities by targeting PAQR3 to activate the PI3K/AKT pathway (Ren et al., 2025; Xiao et al., 2022). Baicalin regulates skin barrier function through the JAK/STAT pathway to improve atopic dermatitis while influencing the gut microbiota to restore metabolic homeostasis (Lin et al., 2022; Wang et al., 2022). Flavonoids and alkaloids from *Sophora alopecuroides* alleviate psoriatic pathological changes through their anti-inflammatory effects while their active metabolite, oxymatrine, also improves lipid metabolism dysregulation by regulating miR-182 (Lin et al., 2024; Zhang et al., 2020). Although individual components have demonstrated multi-target regulatory effects on metabolic and inflammatory abnormalities, the molecular mechanism by which FLM comprehensively improves MS-P comorbidity still requires systematic analysis.

To thoroughly investigate FLM’s mechanism of action, this study employed both apolipoprotein E knockout (APOE^−/−^) and wild-type C57BL/6 mice. APOE^−/−^ mice, lacking the key lipid metabolism regulator ApoE, exhibit phenotypic characteristics that are highly similar to those of human MS under high-fat diet stimulation, including significant insulin resistance, dyslipidemia and chronic inflammation (Zhang et al., 2024). In contrast, wild-type C57BL/6 mice show lower sensitivity to metabolic stress, serving as important controls. By establishing MS-P comorbidity models in mice with different genetic backgrounds, we can comprehensively evaluate FLM’s therapeutic effects and reveal the universality of its mechanisms. This study aims to elucidate how FLM regulates the metabolic-inflammatory network through the miR-29a-5p/IGF-1R signalling axis, providing a theoretical foundation for the development of novel therapeutic strategies.

## 2 Materials and methods

### 2.1 Drugs and reagents

Imiquimod cream was purchased from Med-Shine Pharmaceutical Co., Ltd. (Sichuan; 5%, CFDA approval number: H20030128), and Yinxieling granules were obtained from Pharmaceutical Holding Group Co., Ltd. (Shaanxi; CFDA approval number: Z20010157). ELISA kits, including insulin, granulocyte-macrophage colony stimulating factor (GM-CSF), interferon-γ (IFN-γ), interleukin-9 (IL-9), interleukin-10 (IL-10) and interleukin-17 (IL-17), were purchased from Enzyme-Linked Biotechnology Co., Ltd. (Shanghai). Biochemical assay kits for glucose, total cholesterol (TC), triglyceride (TG) and low-density lipoprotein cholesterol (LDL-C) were obtained from Jiancheng Bioengineering Institute Co., Ltd. (Nanjing). The miR-29a-5p and IGF-1R probes were sourced from Sangon Biotech Co., Ltd. (Shanghai). The *in situ* hybridisation detection kit was purchased from Genfan Biotech Co., Ltd. (Shanghai). IGF-1R antibody was obtained from Abcam Trading Co., Ltd. (Shanghai; ab182408), and β-actin antibody was purchased from Zhongshan Jinqiao Biotechnology Co., Ltd. (Beijing; TA09).

### 2.2 Preparation of Fufang Longdan mixture

FLM consists of *Gentianae Radix Et Rhozima* (10 g), *Scutellariae Radix* (15 g), *Plantaginis Semen* (30 g), *Tetrapanacis Medulla* (6 g), *Sophorae Flavescentis Radix* (15 g), *Tripterygium hypoglaucum* (15 g), *Senecio scandens* (10 g) and *Smilacis Glabrae Rhixoma* (30 g). The mixture was prepared using the traditional water decoction method: the ingredients were extracted twice with an 8-fold volume of purified water under reflux (100°C, 2 h each). The combined extracts were then concentrated under reduced pressure at 60°C until reaching a relative density of 1.05–1.10 (measured at 20°C). Sodium benzoate (0.1%) was added as a preservative, and the preparation was sterilised at 121°C for 15 min after packaging.

### 2.3 UPLC-Q-exactive-MS/MS analysis

FLM samples were extracted with methanol-water (2:1, v/v) and centrifuged before analysis. Separation was performed using a Vanquish UHPLC system (Thermo Scientific, Bremen, Germany) equipped with an ACQUITY UPLC HSS T3 column (2.1 mm × 100 mm, 1.8 μm). The mobile phase consisted of 0.1% formic acid in water (A) and 0.1% formic acid in acetonitrile (B), with a flow rate of 0.3 mL/min. The gradient elution programme progressed from 5% to 98% (B) over 0–20 min. Data were collected using a Q-Exactive HFX mass spectrometer (Thermo Scientific, Bremen, Germany) with electrospray ionisation (ESI) in both positive and negative modes. The full scan/data-dependent MS2 mode was employed, with resolutions of 60,000 and 15,000 for MS1 and MS2, respectively. Data processing was performed using XCMS software, and compounds were identified using the Traditional Chinese Medicine mass spectrometry database (mass error <25 ppm, matching score >0.7).

### 2.4 Model establishment and drug intervention

Male APOE^−/−^ and C57BL/6 mice (6–8 weeks old, 18–22 g, n = 40 each) were purchased from Hunan Slake Jingda Laboratory Animal Co., Ltd. [SCXK(Xiang)2016-0002]. All experimental procedures were approved by the Ethics Committee of Yunnan University of Traditional Chinese Medicine (2022YNU003). Initially, both mouse strains were randomly divided into control (n = 12) and comorbidity (n = 28) groups. The comorbidity groups received a high-fat, high-sugar diet (45% calories from fat, Research Diets, D12451) for 8 weeks, followed by topical application of 5% imiquimod cream (40 mg/d) for 3 weeks starting from week six to induce psoriasis-like changes. The control groups received standard chow (D12450B) and an equivalent petroleum jelly base.

At the end of model establishment, four mice from each group of both strains were randomly selected for model validation. Following successful model validation, the remaining mice in the comorbidity groups (n = 24 per strain) were randomly divided into the model, Yinxieling, and FLM groups (n = 8 per group) for a 4-week intervention period. Yinxueling, a standard clinical treatment for psoriasis, was selected as the positive control. The Yinxueling group received a Yinxueling suspension (8 g/kg/d), calculated from the clinical human dosage (40 g/d) using established body surface area conversion principles (Li et al., 2024). The FLM group received FLM (0.5 mL/d), with this dosage similarly determined through conversion from the standard adult human dosage (100 mL daily) (Luo et al., 2019). The control and model groups received equivalent volumes of saline.

The Psoriasis Area and Severity Index (PASI) score was evaluated weekly using a modified mouse PASI scoring system to assess the level of erythema (0–4), scaling (0–4), skin thickening (0–4) and lesion area (0–6). Scoring was independently performed by two experienced researchers under identical lighting conditions, with the mean values used as the final scores. Blood and skin lesion samples were collected at the experimental endpoint.

### 2.5 Assessment of glucose, insulin and lipid parameters

Fasting glucose was determined using the glucose oxidase method. Serum insulin levels were measured by ELISA following the manufacturer’s protocols. The homeostasis model assessment of insulin resistance (HOMA-IR) was calculated as follows: fasting glucose (mmol/L) × fasting insulin (mIU/L)/22.5. Serum TC, TG and LDL-C levels were measured using an automated biochemical analyser (7600-020, Hitachi, Japan). All samples included internal quality controls, with each parameter measured in triplicate.

### 2.6 Hematoxylin and eosin staining

Fresh lesional tissues were fixed in 4% paraformaldehyde for 24 h, followed by routine dehydration, clearing and paraffin embedding. Sections of 4 μm were prepared using a rotary microtome (RM2125RTS, Leica, Germany). Standard hematoxylin and eosin (H&E) staining was performed, and the sections were examined and photographed using an optical microscope (BX53, Olympus, Japan). The epidermal thickness was measured using ImageJ software, with averages calculated from five random fields per section.

### 2.7 Inflammatory cytokine analysis

Serum levels of GM-CSF, IFN-γ, IL-9, IL-10 and IL-17 were determined via ELISA. All procedures strictly followed the manufacturer’s protocols, with the absorbance measured at 450 nm using a microplate reader (SPECTCA MAX190, Molecular Devices, United States). Each sample was analysed in triplicate, and the concentrations were calculated using standard curves.

### 2.8 Dual-luciferase reporter assay

Luciferase reporter vectors containing wild-type and mutant IGF-1R 3′-UTR sequences (pGL3-IGF-1R-WT/MUT) were constructed. 293T cells (2 × 10^4^ cells/well) were co-transfected with miR-29a-5p mimics or the negative control (NC), along with the corresponding reporter vectors and a pRL-TK reference plasmid. Luciferase activity was measured 48 h post-transfection using a dual-luciferase reporter assay system. Firefly luciferase activity was normalised to *Renilla* luciferase activity. Each experiment was performed in triplicate.

### 2.9 RNA extraction and real-time quantitative PCR

Total RNA was extracted from the lesional tissues using an RNeasy Mini Kit and reverse transcribed using an ExScript RT Reagent Kit. Real-time quantitative PCR was performed using SYBR Premix Ex Taq on a Light Cycle 96 real-time PCR system (Roche, Switzerland). The reaction conditions included denaturation at 95°C for 15 s and annealing at 60°C for 30 s. The expression level of miR-29a-5p was analyzed using U6 as the internal reference gene, while β-actin served as the internal reference gene for IGF-1R expression analysis. The relative expression levels of both targets were calculated using the 2^−ΔΔCt^ method. Each sample was analysed in triplicate, with the experiments repeated three times. The primer sequences are listed in Table 1.

**TABLE 1:** Primer sequences for the target genes.

Gene	Sequence (5′→3′)
miR-29a-5p	F:GCGCGACTGATTTCTTTTGGT
	R:AGTGCAGGGTCCGAGGTATT
IGF-1R	F:CAACTACGCACTGGTCAT
	R:CCGAGTAATGTTCCTCAGATT
U6	F:AGAGAAGATTAGCATGGCCCCTG
	R:AGTGCAGGGTCCGAGGTATT
β-actin	F:TATGGAATCCTGTGGCATC
	R:GTGTTGGCATAGAGGTCTT

### 2.10 Western blot analysis

Lesional tissue samples (100 mg) were lysed in precooled RIPA buffer containing 1% protease inhibitor cocktail using a tissue homogeniser on ice for 30 min. After centrifugation at 12,000 × *g* for 15 min at 4°C, the protein concentrations in the supernatants were determined via BCA assay. Equal amounts of protein were separated by SDS-PAGE using the Mini-PROTEAN system and transferred to PVDF membranes. After blocking with 5% nonfat milk for 2 h at room temperature, the membranes were incubated with IGF-1R antibody (1:1000) or β-actin (1:5000) overnight at 4°C. Following TBST washes, the membranes were incubated with HRP-conjugated secondary antibodies (1:5000) for 1 h at room temperature. Protein bands were visualised using enhanced chemiluminescence ECL and quantified using ImageJ software, with β-actin serving as the loading control. Each experiment was independently repeated three times.

### 2.11 Fluorescence *in situ* hybridisation (FISH)

RNAscope^®^ technology was employed to localise miR-29a-5p and IGF-1R expression in paraffin-embedded lesional tissue sections (4-μm thickness). After deparaffinisation and tissue permeabilisation, hybridisation and signal amplification were performed according to the manufacturer’s protocols. Images were acquired using a laser scanning confocal microscope (LSM800, Zeiss, Germany). Six randomly selected samples per group were analysed for quantification.

### 2.12 Statistical analysis

All data were analysed using SPSS 22.0 software and expressed as the mean ± standard deviation (SD). Group comparisons were made using one-way ANOVA, with *post hoc* Tukey’s test for pairwise comparisons. A p-value of <0.05 was considered statistically significant. Each experiment was independently repeated three times.

## 3 Results

### 3.1 Chemical composition analysis of FLM

#### 3.1.1 Overall distribution of the chemical components

Through UPLC-Q-exactive-MS/MS analysis, we identified 2,665 compounds in FLM, including 1,759 in the positive ion mode and 1,007 in the negative ion mode. Based on the NPClassifier system, these compounds were distributed across eight major categories (Table 2). The shikimates and phenylpropanoids class emerged as the most abundant (793 compounds, 32%), predominantly including flavonoids (283 compounds, 36%), phenolic acids (C6-C1) (98 compounds, 12%) and coumarins (75 compounds, 9%). Alkaloids ranked second (523 compounds, 20%), consisting of tryptophan alkaloids (72 compounds, 14%), anthranilic acid alkaloids (56 compounds, 11%) and pseudoalkaloids (50 compounds, 10%). Among the terpenoids (323 compounds, 13%), the major subclasses were steroids (74 compounds, 23%), monoterpenoids (62 compounds, 19%) and diterpenoids (59 compounds, 18%).

**TABLE 2:** Chemical classification and distribution of the compounds identified in the Fufang Longdan Mixture.

Major class/pathway	Subclass	Count (n)	Percentage (%)
Shikimates and phenylpropanoids (n = 793, 32%)	Flavonoids	283	36
	Phenolic acids(C6-C1)	98	12
	Coumarins	75	9
	Phenylpropanoids(C6-C3)	71	9
	Isoflavonoids	66	8
	Others	200	25
Alkaloids (n = 523, 20%)	Tryptophan alkaloids	72	14
	Anthranilic acid alkaloids	56	11
	Pseudoalkaloids	50	10
	Lysine alkaloids	43	8
	Nicotinic acid alkaloids	40	8
	Others	262	50
Terpenoids (n = 323, 13%)	Steroids	74	23
	Monoterpenoids	62	19
	Diterpenoids	59	18
	Sesquiterpenoids	48	15
	Triterpenoids	41	13
	Others	39	12
Fatty acids (n = 241, 10%)	Fatty acids and their conjugates	101	42
	Octadecanoids	30	12
	Fatty esters	24	10
	Fatty acyls	17	7
	Fatty amides	17	7
	Others	52	22
Amino and peptide acids (n = 238, 10%)	Small peptides	224	94
	Others	14	6
Polyketides (n = 187, 8%)	Polycyclic aromatic polyketides	34	18
	Aromatic polyketide	26	14
	Chromanes	24	13
	Naphthalenes	16	9
	Cyclic polyketide	11	6
	Others	76	41
Carbohydrates (n = 75, 3%)	Saccharides	38	51
	Nucleosides	28	37
	Aminosug and aminoglycosides	7	9
	Others	2	3
Others (n = 87, 4%)	Meroterpenoids	14	16
	Peptides alkaloids	9	10
	Phenylpropanoids(C6-C3)	7	8
	Pseudoalkaioids	6	7
	Small peptides	6	7
	Others	45	52

#### 3.1.2 Characteristic peak analysis and compound identification

**FIGURE 1:**
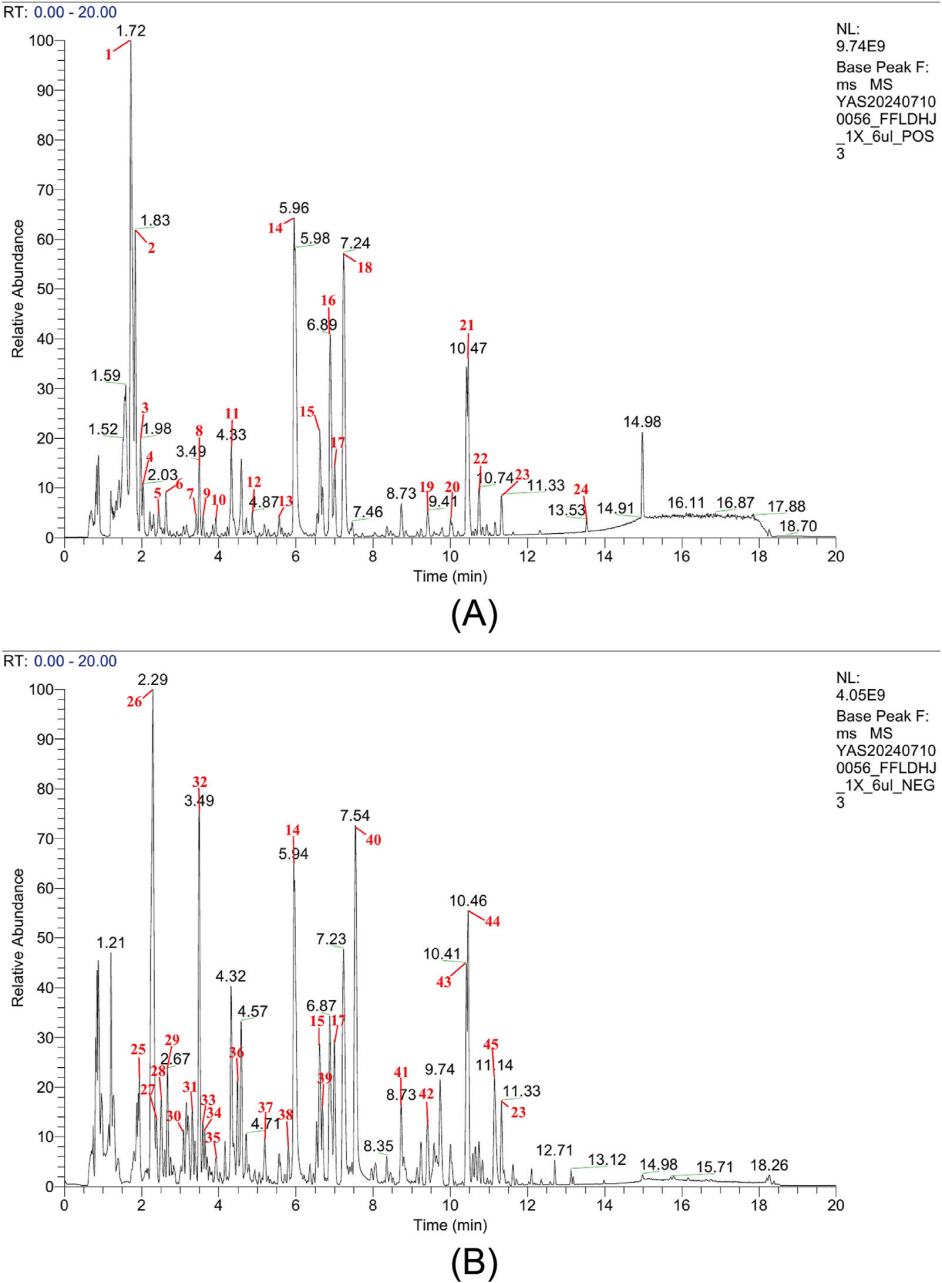
Chemical analysis of the Fufang Longdan Mixture (FLM) via UPLC-Q-exactive-MS/MS. **(A)** Base peak chromatogram in the positive ion mode showing characteristic peaks. **(B)** Base peak chromatogram in the negative ion mode showing characteristic peaks. Numbers correspond to the compounds listed in Supplementary Table S1. NL, normalisation level.

The base peak chromatogram analysis revealed 45 characteristic compound peaks in the FLM (Figure 1), with the corresponding identification details listed in Supplementary Table S1. In the positive ion mode, the prominent characteristic peaks included marine (RT = 1.73 min, [M + H]+ m/z 249.196), baicalin (RT = 5.97 min, [M + H]+ m/z 447.0917) and oroxindin (RT = 6.89 min, [M + H]+ m/z 461.1075). Significant peaks in the negative ion mode comprised chlorogenate (RT = 3.08 min, [M-H]- m/z 353.0879) and baicalein (RT = 8.74 min, [M-H]- m/z 269.0455).

### 3.2 Validation of the MS-P comorbidity model

#### 3.2.1 Metabolic parameter changes

**FIGURE 2:**
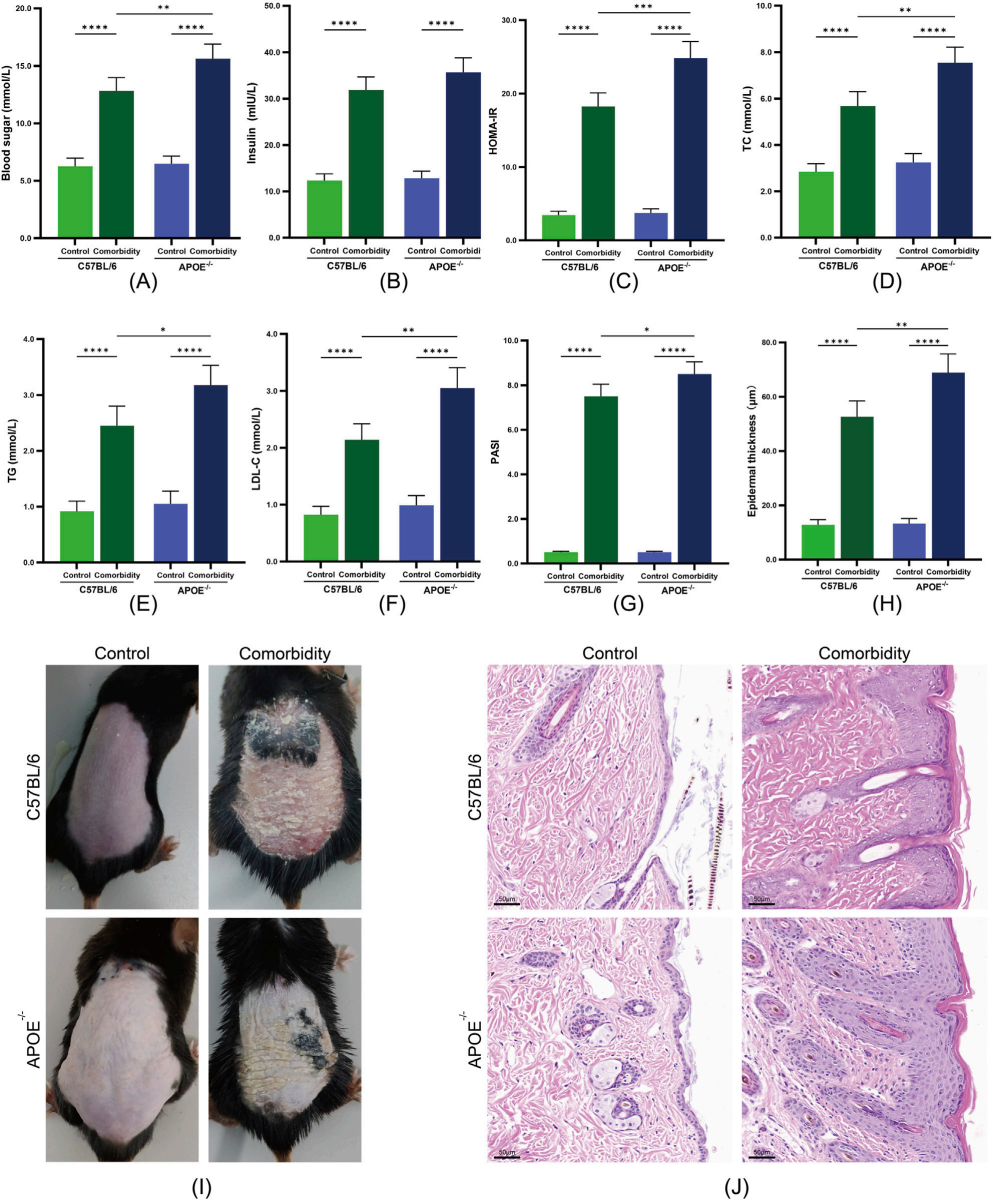
Validation of the metabolic syndrome and psoriasis (MS-P) comorbidity model in APOE^−/−^ and C57BL/6 mice. **(A)** Blood glucose levels. **(B)** Serum insulin levels. **(C)** HOMA-IR index. **(D)** Total cholesterol (TC) levels. **(E)** Triglycerides (TG) levels. **(F)** LDL-C levels. **(G)** Psoriasis Area and Severity Index (PASI) scores. **(H)** Epidermal thickness quantification. **(I)** Representative macroscopic images of dorsal skin lesions. **(J)** Representative H&E staining showing epidermal hyperplasia (scale bar = 50 μm). Statistical significance notation across all figures: *p < 0.05, **p < 0.01, ***p < 0.001, ****p < 0.0001.

We successfully established an MS-P comorbidity model through a high-fat, high-sugar diet combined with imiquimod-induced psoriasis-like lesions. Both APOE^−/−^ and C57BL/6 mice in the comorbidity groups demonstrated significant metabolic disruption compared with their respective control groups. Among the metabolic parameters, marked elevation was observed in fasting blood glucose and the HOMA-IR index (Figures 2A–C). The lipid profile analyses revealed significant increases in the TC, triglycerides and LDL-C levels (Figures 2D–F).

Notably, the response to model induction varied between the two mouse strains. The APOE^−/−^ comorbidity group exhibited more severe metabolic disruption, with significantly higher levels of blood glucose, HOMA-IR, TC, TG and LDL-C compared with the C57BL/6 comorbidity group. This differential response suggests that APOE gene deficiency may exacerbate the progression of metabolic dysfunction.

#### 3.2.2 Development of psoriasis-like lesions

The comorbidity groups exhibited characteristic psoriasis-like changes. Macroscopic examination revealed scale formation and erythema, with significantly elevated PASI scores compared with the control groups (Figures 2G–I). Histopathological examination revealed marked epidermal thickening, hyperkeratosis with parakeratosis, numerous Munro microabscesses and dermal inflammatory cell infiltration (Figure 2J). APOE^−/−^ mice exhibited significantly higher PASI scores compared to C57BL/6 mice, suggesting that APOE gene deficiency may intensify the dysregulation of the inflammation-metabolism network. These results confirmed our successful establishment of MS-P comorbidity models with different genetic backgrounds, providing a foundation for subsequent therapeutic evaluation.

### 3.3 Therapeutic effects of FLM on MS-P comorbidity

#### 3.3.1 Regulation of metabolic dysfunction

**FIGURE 3:**
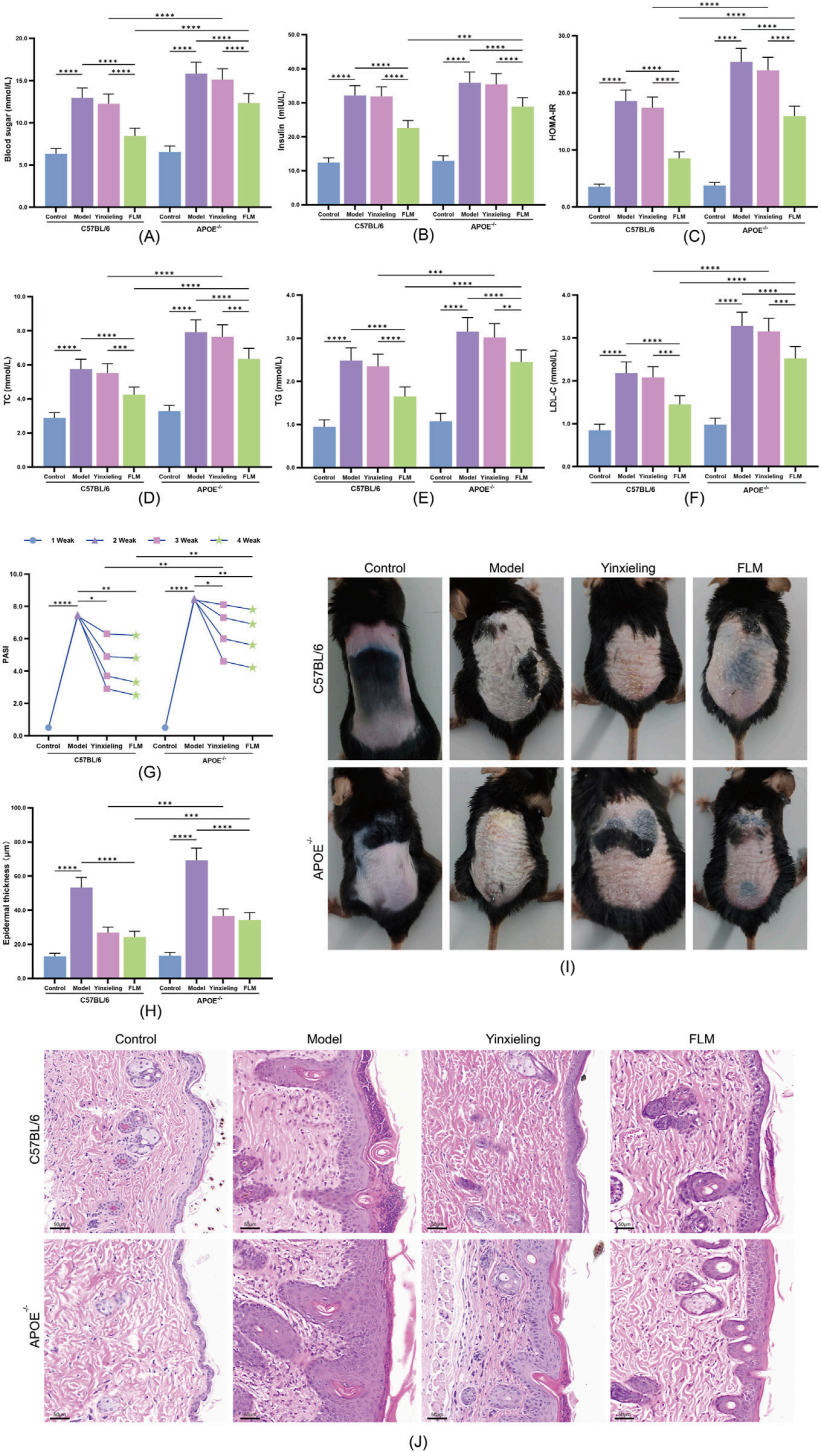
Therapeutic effects of FLM on metabolic parameters and skin lesions in APOE^−/−^ and C57BL/6 mice. **(A–F)** Metabolic parameters including blood glucose, insulin, HOMA-IR, TC, TG, and LDL-C levels. **(G)** Weekly PASI scores during treatment. **(H)** Epidermal thickness quantification. **(I)** Representative macroscopic images of dorsal skin lesions at week 4. **(J)** Representative H&E staining showing epidermal changes (scale bar = 50 μm). Statistical significance notation across all figures: *p < 0.05, **p < 0.01, ***p < 0.001, ****p < 0.0001.

Following 4 weeks of drug intervention, the administration of FLM significantly improved the metabolic parameters in both mouse strains. In C57BL/6 mice, the FLM group showed significantly reduced fasting blood glucose and HOMA-IR index scores compared with the model group (Figures 3A–C). The lipid parameters also improved significantly, as evidenced by decreased TC, TG and LDL-C levels (Figures 3D–F). While APOE^−/−^ mice also responded to the treatment, the improvement magnitude was smaller. Notably, the Yinxieling group exhibited no significant improvement in metabolic parameters in either mouse strain, highlighting FLM’s unique advantage in metabolic regulation.

#### 3.3.2 Improvement of skin lesions

The improvement of skin lesions demonstrated clear time- and genotype-dependent patterns. C57BL/6 mice showed significant PASI score reduction after 2 weeks of treatment (Figure 3G), reaching maximum improvement at 4 weeks. Macroscopic examination (Figure 3I) and H&E staining (Figure 3J) revealed marked inhibition of epidermal hyperplasia, with significant reduction in hyperkeratosis and inflammatory cell infiltration. APOE^−/−^ mice showed a delayed treatment response, requiring over 3 weeks of treatment for significant PASI score improvement, as confirmed by quantitative analysis of epidermal thickness (Figure 3H). Regarding skin lesion improvement, the Yinxieling group showed comparable improvement to FLM, consistent with its clinical positioning.

### 3.4 Anti-inflammatory mechanism of FLM

**FIGURE 4:**
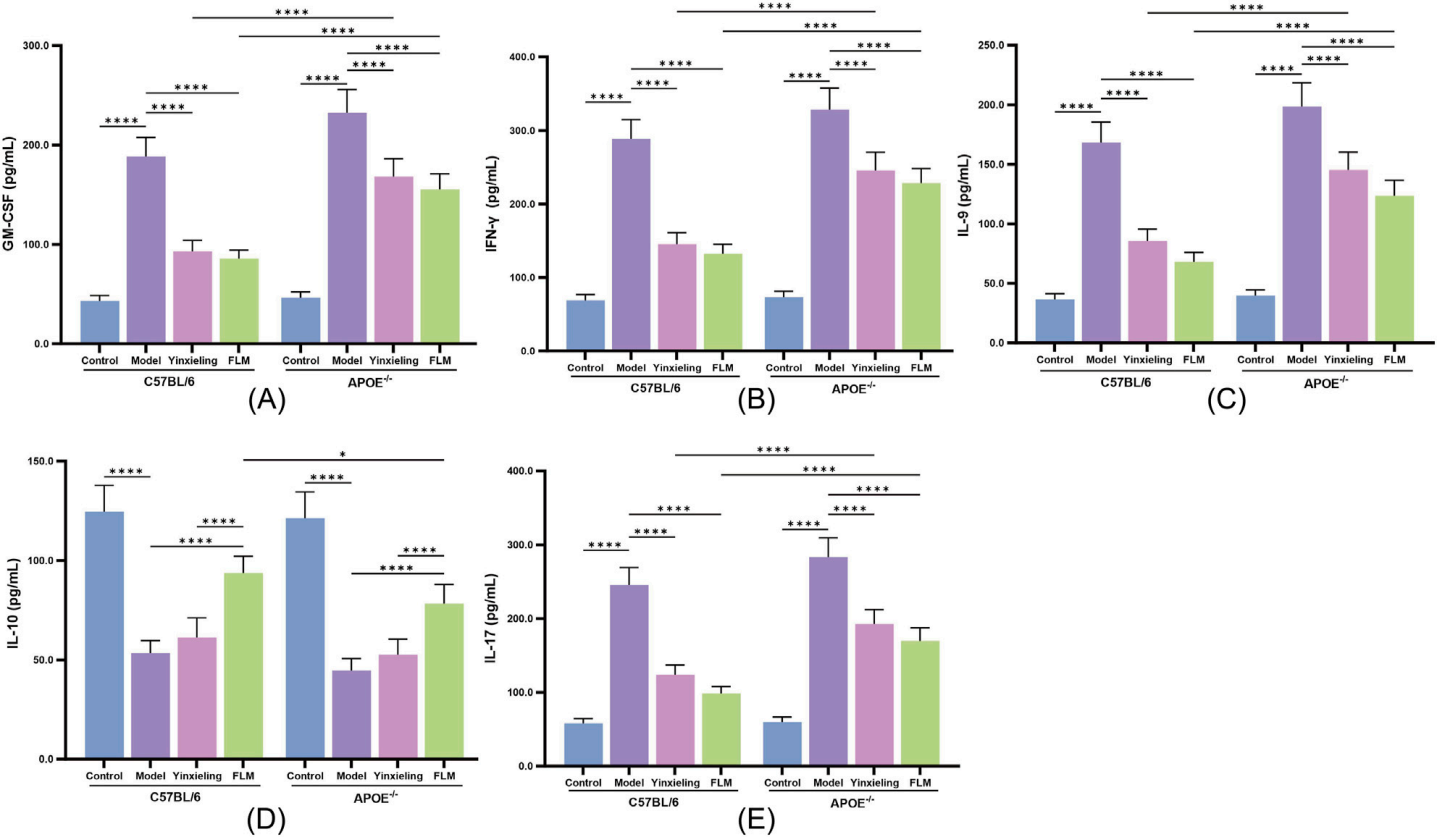
Effects of FLM on inflammatory cytokine profiles in APOE^−/−^ and C57BL/6 mice. **(A)** GM-CSF levels. **(B)** IFN-γ levels. **(C)** IL-9 levels. **(D)** IL-10 levels. **(E)** IL-17 levels. Statistical significance notation across all figures: *p < 0.05, **p < 0.01, ***p < 0.001, ****p < 0.0001.

FLM exhibited significant immunomodulatory effects. In C57BL/6 mice, the treatment significantly reduced the expression of the pro-inflammatory cytokines GM-CSF, IFN-γ, IL-9 and IL-17, while simultaneously elevating the anti-inflammatory IL-10 levels (Figures 4A–E). APOE^−/−^ mice showed similar but less pronounced regulatory trends. Compared with Yinxieling, FLM not only exhibited inhibitory effects on pro-inflammatory cytokines but also demonstrated additional advantages in promoting anti-inflammatory cytokine expression, reflecting its comprehensive immunomodulatory characteristics.

### 3.5 Regulatory effects on the miR-29a-5p/IGF-1R signalling axis

#### 3.5.1 Validation of direct targeting of IGF-1R by miR-29a-5p

**FIGURE 5:**
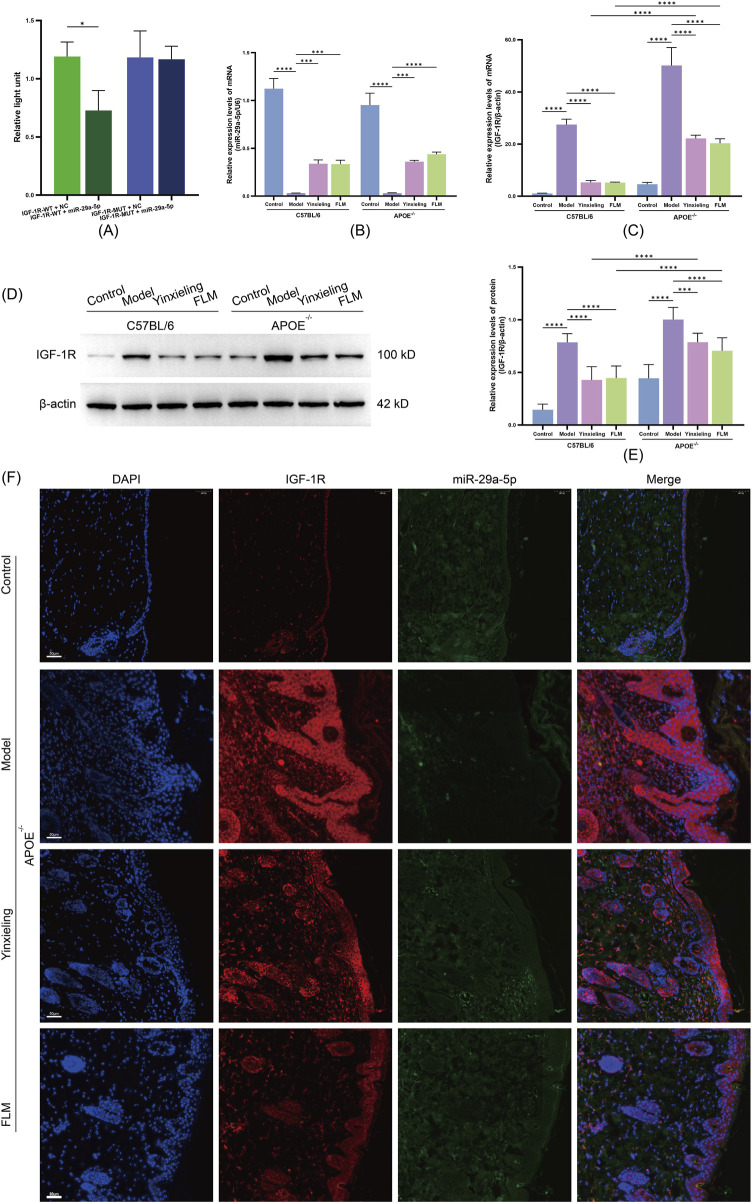
Analysis of miR-29a-5p/IGF-1R axis regulation by FLM in APOE^−/−^ and C57BL/6 mice. **(A)** Dual-luciferase reporter assay of IGF-1R 3′-UTR activity. **(B)** Relative miR-29a-5p expression by RT-qPCR. **(C)** IGF-1R mRNA expression by RT-qPCR. **(D)** Western blot analysis of IGF-1R protein levels. **(E)** Quantification of IGF-1R protein expression. **(F)** FISH analysis showing co-localisation of miR-29a-5p (green) and IGF-1R (red) in skin lesions. DAPI (blue) indicates nuclei. Scale bar = 50 μm. Statistical significance notation across all figures: *p < 0.05, **p < 0.01, ***p < 0.001, ****p < 0.0001.

To verify IGF-1R as a direct target gene of miR-29a-5p, we constructed dual-luciferase reporter vectors containing wild-type and mutant IGF-1R 3′-UTR sequences. Our results showed that compared with the negative control group (IGF-1R-WT + NC), miR-29a-5p overexpression (IGF-1R-WT + miR-29a-5p group) significantly reduced the relative luciferase activity of the wild-type IGF-1R 3′-UTR. In contrast, miR-29a-5p overexpression (IGF-1R-MUT + miR-29a-5p group) had no significant effect on luciferase activity in the mutant IGF-1R 3′-UTR group (Figure 5A). These results confirmed that miR-29a-5p directly regulates IGF-1R expression through specific recognition and binding to its 3′-UTR sequence.

#### 3.5.2 Effects on miR-29a-5p and IGF-1R expression

To elucidate the molecular mechanism by which FLM improves metabolic-inflammatory network dysregulation, we analysed the characteristics of the miR-29a-5p/IGF-1R signalling axis at the gene transcription, protein expression and tissue localisation levels. RT-qPCR analysis revealed that compared with the control groups, both mouse strains in the model groups showed significantly decreased miR-29a-5p expression and increased IGF-1R mRNA levels (Figures 5B,C). FLM intervention effectively reversed this expression pattern, with significant upregulation of miR-29a-5p and downregulation of IGF-1R mRNA observed in both C57BL/6 and APOE^−/−^ mice, with more pronounced effects observed in C57BL/6 mice. Western blot analysis confirmed that the IGF-1R protein level changes corresponded with its mRNA expression pattern, with FLM treatment significantly reducing elevated IGF-1R protein expression in both mouse models (Figures 5D,E).

#### 3.5.3 Tissue localisation and interaction of miR-29a-5p/IGF-1R

Using fluorescence *in situ* hybridisation, we observed the spatial distribution characteristics of miR-29a-5p and IGF-1R expression in the lesional tissues. The model groups showed significantly decreased miR-29a-5p expression (reduced FAM fluorescence signal) and increased IGF-1R expression (enhanced CY3 fluorescence signal) compared with the control groups. After FLM intervention, the lesional tissues showed significantly restored miR-29a-5p expression levels and notably reduced IGF-1R expression, with evident co-localisation at the cellular level (Figure 5F). This regulatory effect was more pronounced in the C57BL/6 mice. These results demonstrate that FLM exerts its therapeutic effects through the regulation of the miR-29a-5p/IGF-1R axis, with this regulatory effect showing clear genotype dependence.

## 4 Discussion

The comorbidity of metabolic syndrome and psoriasis (MS-P) significantly increases the disease burden and treatment complexity (Hao et al., 2021; Rong et al., 2024). Previous research has primarily focused on the pathogenesis of the individual conditions, making it challenging to understand their interactions (Sieminska et al., 2024). Traditional animal models typically address single disease characteristics, failing to accurately simulate the complex metabolic-inflammatory network dysregulation in comorbid states. Our study employed a combined approach using a high-fat, high-sugar diet coupled with imiquimod-induced local stimulation to establish a comorbidity model. Imiquimod, an FDA-approved topical medication for treating external genital warts, induces psoriasis-like lesions characterised by epidermal thickening, scaling and erythema (Dika et al., 2018). Using this combined modelling strategy, we successfully established an animal model exhibiting metabolic-inflammatory network dysregulation. The model mice demonstrated both significant metabolic abnormalities, including elevated fasting glucose, insulin resistance index scores and disrupted lipid profiles and typical dorsal lesions, characterised by increased PASI scores and epidermal hyperplasia. This combined modelling approach provides a reliable experimental platform for investigating comorbidity mechanisms.

As a multi-component complex system, FLM has long faced challenges in elucidating its chemical basis and mechanisms of action. Our UPLC-Q-exactive-MS/MS analysis revealed abundant flavonoids, alkaloids and terpenoids in the FLM. Our animal experiments demonstrated FLM’s significant advantages in improving comorbid conditions. Compared with the traditional antipsoriatic drug, Yinxieling, FLM not only improved skin lesions and metabolic parameters but also significantly modulated inflammatory cytokine expression. This multi-target efficacy likely stems from the synergistic effects of the multiple active components. Oroxindin reduces inflammatory cascade reactions by inhibiting NLRP3 inflammasome activation (Liu et al., 2020), oxymatrine improves psoriatic pruritus and inflammation by downregulating HSP90/HSP60 expression in keratinocytes (Xiang et al., 2020), and dehydrocorydaline exerts anti-inflammatory and analgesic functions through transient receptor potential (TRP) channels (Zeng et al., 2025). Wogonin improves lipid metabolism abnormalities by activating the PPARα/AdipoR2 signal (Chen et al., 2017), whereas baicalein alleviates hepatic lipid deposition by restoring the fatty acid synthesis and oxidation metabolism balance (Li et al., 2022). These findings systematically reveal FLM’s ‘component-mechanism-effect’ mapping relationship, establishing a molecular foundation for understanding its comprehensive therapeutic network.

In evaluating the therapeutic efficacy of FLM, we observed striking phenotypic variations and differential treatment responses across mice with distinct genetic backgrounds. The impact of APOE gene deficiency on disease progression and therapeutic outcomes aligns with previous findings (Lin et al., 2025; Tu et al., 2015). For instance, Xu et al. demonstrated that APOE^−/−^ mice exhibit heightened sensitivity to metabolic stress, manifesting more severe insulin resistance and lipid metabolism disorders (Xu et al., 2021). Jin et al. further established that APOE deficiency exacerbates high-fat diet-induced systemic inflammation, characterised by significantly elevated pro-inflammatory cytokine levels and intensified tissue inflammatory infiltration (Jin et al., 2016). Notably, our study revealed that APOE^−/−^ mice showed markedly attenuated responses to FLM intervention, with diminished improvement in both metabolic parameters and delayed resolution of skin lesions. These findings not only deepen our understanding of APOE’s role in MS-P pathogenesis but also provide crucial insights for developing personalised therapeutic strategies.

To elucidate the molecular mechanisms underlying FLM’s therapeutic effects on MS-P comorbidity, we employed a multi-tiered experimental approach to systematically validate the regulatory relationship between miR-29a-5p and IGF-1R. Initially, dual-luciferase reporter assays confirmed IGF-1R as a direct target of miR-29a-5p, establishing the molecular foundation for subsequent investigations. We further validated this regulatory relationship at the transcriptional and protein levels through RT-qPCR and Western blot analyses. Moreover, fluorescence *in situ* hybridisation revealed the co-localisation of miR-29a-5p and IGF-1R at the tissue level. This hierarchical validation strategy, spanning from the molecular level to the tissue level, not only substantiated the direct regulatory effect of miR-29a-5p on IGF-1R but also provided robust experimental evidence for understanding FLM’s therapeutic mechanism. Significantly, we observed that APOE^−/−^ mice exhibited substantially lower miR-29a-5p expression levels compared with wild-type mice, accompanied by relatively elevated IGF-1R expression. This finding suggests that APOE may participate in MS-P pathogenesis through the modulation of the miR-29a-5p/IGF-1R axis, offering new molecular mechanistic insights into how genetic background can influence therapeutic outcomes.

## 5 Conclusion

Through comprehensive UPLC-Q-exactive-MS/MS analysis, we systematically characterised the chemical composition of FLM, identifying 2,665 distinct compounds, with flavonoids, alkaloids and terpenoids being the predominant bioactive constituents, thereby establishing a solid foundation for mechanistic investigation. Building upon this chemical characterisation, we successfully developed an MS-P comorbidity model in both APOE^−/−^ and C57BL/6 mice using a combined approach of a high-fat, high-sugar diet and imiquimod induction. Notably, APOE^−/−^ mice exhibited markedly intensified metabolic dysfunction and inflammatory responses, coupled with attenuated therapeutic responsiveness to FLM intervention, highlighting the significant impact of genetic background on disease progression and treatment efficacy. Most significantly, through multi-level molecular mechanistic studies, we demonstrated that IGF-1R serves as a direct target of miR-29a-5p and elucidated that FLM exerts its therapeutic effects by upregulating miR-29a-5p expression, thereby suppressing IGF-1R expression. These findings not only provide robust experimental evidence supporting FLM’s therapeutic potential in treating MS-P comorbidity but also offer novel insights for the development of targeted therapeutic strategies.

## Data Availability

The original contributions presented in the study are included in the article/Supplementary Material, further inquiries can be directed to the corresponding authors.
