# Supplementary material for: Therapeutic effects and mechanisms of Fufang Longdan mixture on metabolic syndrome with psoriasis via miR-29a-5p/IGF-1R axis
Source: Front Pharmacol. 2025 May 9;16:1585369. doi: 10.3389/fphar.2025.1585369 (PMC12098636; doi:10.3389/fphar.2025.1585369)
Supplement: Supplementary file 1 [file Table1.pdf]

Therapeutic Effects and Mechanisms of Fufang Longdan Mixture on Metabolic Syndrome with Psoriasis via miR-29a-5p/IGF-1R Axis

Guangyun Luo[ab†], Xiangyi Kong[b†], Fang Wang[c], Zhiming Wang[b], Zhuo Zhang[b], Huan Cui[b], Yiwen Zhang[b], Wen Huang[b], Xuesong Yang[b*], Jianzhou Ye[ab*]

[a] Nanjing University of Chinese Medicine, Nanjing, Jiangsu 210000, China;

[b] Yunnan University of Chinese Medicine, Kunming, Yunnan 650032, China;

[c] Department of Dermatology, First Affiliated Hospital of Yunnan University of Chinese Medicine, Kunming 650032, Yunnan, China

[†]These authors contributed equally to this work

[*]Corresponding authors at: JianzhouYe; XuesongYang, Nanjing University of Chinese Medicine, Nanjing, China; E-mail addresses: trcm5258@163.com (J. Ye); yangxuesong@ynucm.edu.cn(X.Yang).

Table S1. Identification of characteristic peaks in BPC chromatograms of Fufang Longdan Mixture

No.	Compound Name	m/z	RT/min	ppm	Adduct	Score	Chemical Class
1	Matrine	249.1960	1.73	0.6	[M+H]+	0.9999	Lysine alkaloids
2	Sophocarpine	247.1810	1.84	0.3	[M+H]+	0.9998	Lysine alkaloids
3	N-Acetyl-L-phenylalanine	166.0860	1.98	0.3	[M+H-C2H2O]+	0.9989	Small peptides
4	5-Ureidovaleric acid	100.0760	2.04	1.1	[M+H-CH3NO2]+	0.9998	Small peptides
5	DL-Ephedrine	166.1230	2.44	0.5	[M+H]+	0.9978	Pseudoalkaloids
6	Tryptophan	188.0710	2.64	1.1	[M+H-NH3]+	0.9677	Small peptides
7	Pentobarbital	227.1390	3.41	0.9	[M+H]+	0.9259	Peptide alkaloids
8	Gibepyrone D	195.0650	3.50	1.7	[M+H]+	0.9685	Cyclic polyketides+Monoterpenoids
9	Grandifloroside	197.0810	3.59	0.8	[M+H-C15H18O9] +	0.9862	Monoterpenoids
10	Ethirimol	182.1290	3.93	0.5	[M+H-C2H4]+	0.9655	NA
11	Chrysin 6-C-glucoside 8-C-arabinoside	549.1600	4.33	0.4	[M+H]+	0.9564	Flavonoids
12	Sipeimine	430.3315	4.88	0.9	[M+H]+	0.9984	Pseudoalkaloids
13	Majarine	336.1231	5.57	1.2	[M+]+	0.993	Tyrosine alkaloids
14	Baicalin	447.0917	5.97	1.4	[M+H]+	0.9997	Flavonoids
15	Genistein-4'-glucuronide	447.0919	6.62	0.9	[M+H]+	0.9984	Isoflavonoids
16	Oroxindin	461.1075	6.89	1.0	[M+H]+	0.9982	Flavonoids
17	Hispidulin 7-glucuronide	477.1025	7.01	1.0	[M+H]+	0.9965	Flavonoids
18	Oroxyloside	461.1076	7.25	0.4	[M+H]+	0.993	Flavonoids
19	Formononetin	269.0808	9.43	0.3	[M+H]+	0.9958	Isoflavonoids
20	Isoxanthohumol	355.1539	10.01	1.7	[M+H]+	0.9929	Flavonoids
21	Neobaicalein	375.1073	10.47	0.9	[M+H]+	0.995	Flavonoids
22	Wogonin	285.0757	10.74	0.5	[M+H]+	0.999	Flavonoids
23	Kurarinone	439.2114	11.33	0.3	[M+H]+	0.974	Flavonoids

24	2,3',4,5'-Tetramethoxystilbene	301.1408	13.54	9.0	[M+H]+	0.9996	Stilbenoids
25	trans-1,4-Cyclohexanedicarboxylic acid	171.0660	1.95	0.5	[M-H]-	0.8386	NA
26	1,3-Dimethyl-2-hydroxythioxanthone	255.0510	2.29	9.5	[M-H]-	0.75	NA
27	Benzoic acid + 2O, O-Hex	315.0723	2.39	6.6	[M-H]-	0.9801	Phenolic acids (C6-C1)
28	Neochlorogenic acid	353.0878	2.51	0.4	[M-H]-	0.9476	Phenylpropanoids (C6-C3)
29	Loganic acid	375.1296	2.68	0.5	[M-H]-	0.9014	Monoterpenoids
30	Chlorogenate	353.0879	3.08	0.6	[M-H]-	0.9956	Phenylpropanoids (C6-C3)
31	Acetylsyringic acid	239.0562	3.32	0.8	[M-H]-	0.8136	Phenolic acids (C6-C1)
32	Gentiopicroside	401.1087	3.50	0.0	[M+FA-H]-	0.8087	Monoterpenoids
33	Sweroside	403.1246	3.60	0.2	[M+HCO2]-	0.9838	Monoterpenoids
34	Caffeate	179.0349	3.64	0.7	[M-H]-	0.999	Phenylpropanoids (C6-C3)
35	2,3-Dihydroxybenzoic acid	153.0194	3.93	0.7	[M-H]-	0.9983	Phenolic acids (C6-C1)
36	Coumaric acid	163.0400	4.49	0.8	[M-H]-	0.9981	Phenylpropanoids (C6-C3)
37	Kaempferol-3-o-glucoside	447.0934	5.19	1.3	[M-H]-	0.9862	Flavonoids
38	Azelaic acid	187.0976	5.81	0.8	[M-H]-	0.9973	Fatty Acids and Conjugates
39	6-O-Methylscutellarin	475.0882	6.69	0.8	[M-H]-	0.9397	Flavonoids
40	Ethyl 3-hydroxybenzoate	165.0556	7.54	0.4	[M-H]-	0.9926	Phenolic acids (C6-C1)
41	Baicalein	269.0455	8.74	0.8	[M-H]-	0.9997	Flavonoids
42	7-Hydroxy-3-(2-methoxyphenyl)coumarin	267.0662	9.42	0.2	[M-H]-	0.9961	Coumarins
43	Calycosin	283.0610	10.43	1.3	[M-H]-	0.9957	Isoflavonoids
44	5-Hydroxy-2-(3-hydroxy-4,5-dimethoxyphenyl)-3,7-dimethoxychromone	373.0927	10.47	0.9	[M-H]-	0.9122	Flavonoids
45	Peucenin	259.1009	11.16	12.0	[M-H]-	0.9901	Chromanes

Note: (Column header explanations: NO: Number; m/z: mass-to-charge ratio of parent ion; RT/min: retention time in minutes; ppm: mass accuracy of MS1; Adduct: adducted form; Score: MS2 spectral matching score; Compound EN: compound name in English; Compound CN: compound name in Chinese; SuperClass: compound classification; NA: not available; *compounds identified using reference standards.)
