# Supplementary material for: Therapeutic effects and mechanisms of Fufang Longdan mixture on metabolic syndrome with psoriasis via miR-29a-5p/IGF-1R axis
Source: Front Pharmacol. 2025 May 9;16:1585369. doi: 10.3389/fphar.2025.1585369 (PMC12098636; doi:10.3389/fphar.2025.1585369)
Supplement: Supplementary file 2 [file DataSheet1.pdf]

Therapeutic Effects and Mechanisms of Fufang Longdan Mixture on Metabolic Syndrome with Psoriasis via miR-29a-5p/IGF-1R Axis

Guangyun Luo[ab†], Xiangyi Kong[b†], Fang Wang[c], Zhiming Wang[b], Zhuo Zhang[b], Huan Cui[b], Yiwen Zhang[b], Wen Huang[b], Xuesong Yang[b*], Jianzhou Ye[ab*]

[a] Nanjing University of Chinese Medicine, Nanjing, Jiangsu 210000, China;

[b] Yunnan University of Chinese Medicine, Kunming, Yunnan 650032, China;

[c] Department of Dermatology, First Affiliated Hospital of Yunnan University of Chinese Medicine, Kunming 650032, Yunnan, China

[†]These authors contributed equally to this work

[*]Corresponding authors at: JianzhouYe; XuesongYang, Nanjing University of Chinese Medicine, Nanjing, China; E-mail addresses: trcm5258@163.com (J. Ye); yangxuesong@ynucm.edu.cn(X.Yang).

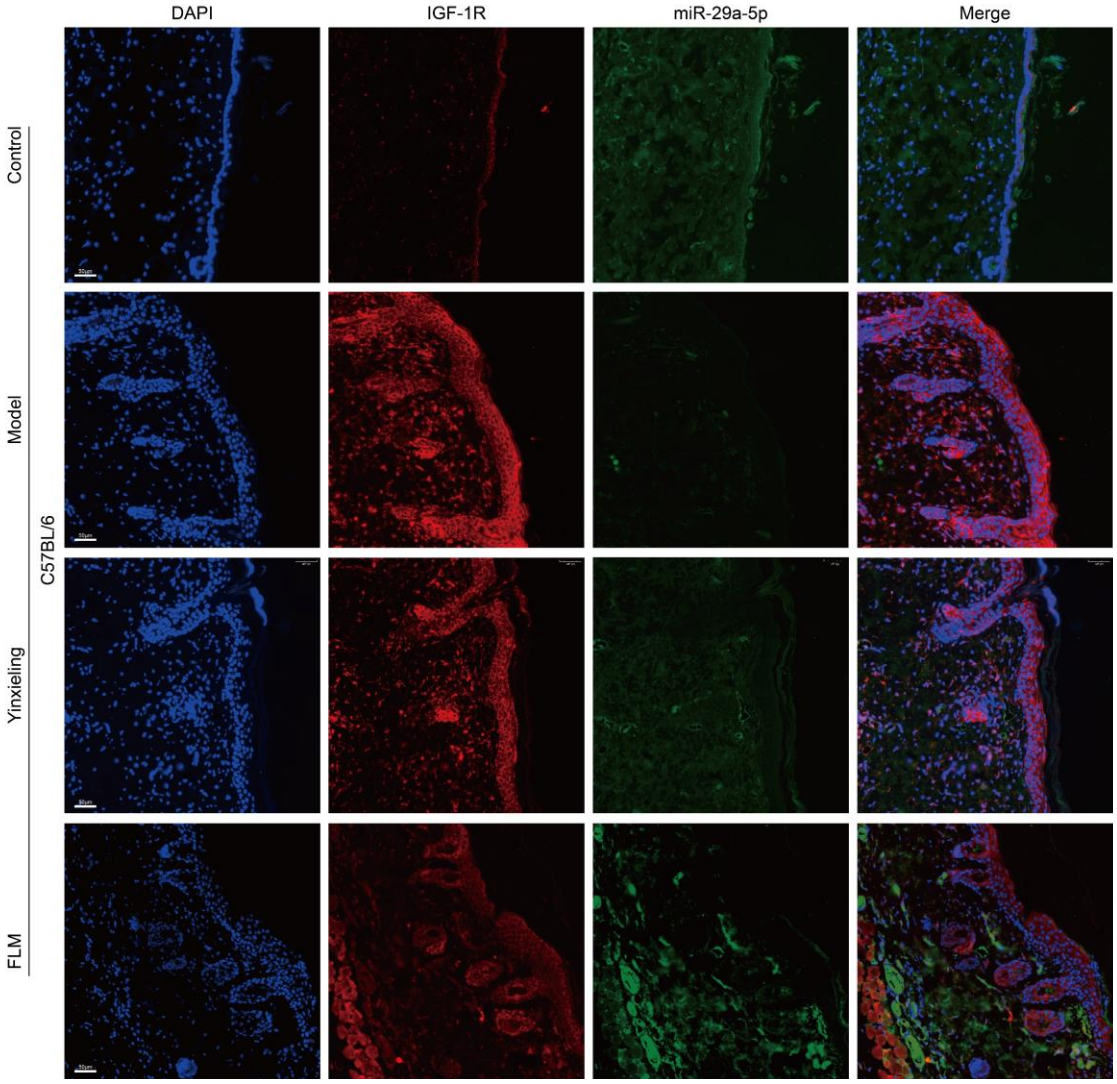

Figure S1. Additional FISH analysis in C57BL/6 mouse skin lesions showing co-localization of miR-29a-5p and IGF-1R in different treatment groups.
